# Factors Associated with Suicidal Ideation and Suicide Attempt among School-Going Urban Adolescents in Peru

**DOI:** 10.3390/ijerph121114842

**Published:** 2015-11-20

**Authors:** Bimala Sharma, Eun Woo Nam, Ha Yun Kim, Jong Koo Kim

**Affiliations:** 1 Yonsei Global Health Center, Yonsei University, 1 Yonseidae-gil, Wonju City, Gangwon-do, 220-710, Korea; E-Mails: bimalasharma@gmail.com (B.S.); khy9053@naver.com (H.Y.K.); kimjk214@yonsei.ac.kr (J.K.K.); 2 Institute for Poverty Alleviation and International Development, Yonsei University, 1 Yonseidae-gil, Wonju City, Gangwon-do, 220-710, Korea; 3 Department of Health Administration, Graduate School, Yonsei University, 1 Yonseidae-gil, Wonju City, Gangwon-do, 220-710, Korea; 4 Department of Family Medicine, Wonju College of Medicine, Yonsei University, Wonju City, Gangwon-do, 220-710, Korea

**Keywords:** suicidal ideation, suicide attempt, factors, adolescents, Peru

## Abstract

The study examines the prevalence of suicidal ideation and suicide attempt, and associated factors among school-going urban adolescents in Peru. A cross-sectional survey was conducted in a sample of 916 secondary school adolescents in 2014. A structured questionnaire adapted from Global School-based Student Health Survey was used to obtain information. Data were analyzed using logistic regression models at 5% level of significance. Overall, 26.3% reported having suicidal ideation, and 17.5% reported having attempted suicide during the past 12 months. Multivariate logistic regression analysis showed that female sex, being in a fight, being insulted, being attacked, perceived unhappiness, smoking and sexual intercourse initiation were significantly associated with increased risk of suicidal ideation, while female sex, being in a fight, being insulted, being attacked, perceived unhappiness, alcohol and illicit drug use were related to suicide attempt. The prevalence of suicidal ideation and suicide attempts observed in the survey area is relatively high. Female adolescents are particularly vulnerable to report suicidal ideation and suicide attempt. Interventions that address the issue of violence against adolescents, fighting with peers, health risk behaviors particularly initiation of smoking, alcohol and illicit drug use and encourage supportive role of parents may reduce the risk of suicidal behaviors.

## 1. Introduction

Suicide is a global public health issue. More than 800,000 people commit suicide and many more attempt it every year [1]. In 2012 suicide accounted for 1.4% of all deaths and was the 15th leading cause of death worldwide [2]. Moreover, suicide was the fourth leading cause of death among young males and the third among young females aged 15–19 years [3]. The Global Burden of Disease Study 2004 estimated that suicide contributes to 6% of all deaths among young people aged 10–24 years worldwide [4]. Notably, 75% of global suicides occur in low- and middle-income countries [1,2]. Previously, suicide rates were found to be low in Latin American countries, but that has been expected to change [5]. In Peru, the suicide rates for males and females were 1.1 and 0.6 per 100,000 population and among all persons treated in mental health outpatient facilities in 2011, 33% were younger than 18 years [6]. Lifetime suicidal thoughts among women of reproductive age were reported to be 28.7% [7]. As very few studies have been conducted in Peru on suicidal behavior among adolescents, the prevalence of suicidal ideation, suicide attempt and the risk factors in the adolescent population remain unclear.

Suicide is a serious public health problem, but it is preventable with timely and low-cost intervention [1]. To date, several environmental, psychosocial and behavioral factors have been found to be associated with suicidal ideation, suicidal attempts and suicide in studies conducted in various countries. Physical, sexual and emotional abuse has been associated with adolescent suicidal ideation and attempts [8,9,10]. In addition, childhood physical and sexual abuse appears to be risk factors for future suicide attempts [11]. It is noted that suicidal behaviors often coexist with other health risk behaviors [12,13]. Studies have shown that aggressive behavior [12], smoking [12], illicit drug use [14], alcohol use [15] and experience of sexual intercourse [16] are associated with suicidal behaviors in adolescents. Studies in other countries also revealed that parental support, family factors as well as psychological factors also influence suicidal behavior [14,16]. Such findings suggest that recognizing health risk behaviors [13] and other factors that influence suicidal behavior can improve understanding about those at high risk of suicide. By reviewing the previous studies conducted in different countries, it is observed that prevalence of suicidal ideation in a year varies widely, ranging approximately from 8% to 30% [14,15,16,17].

Suicidal ideation is both a strong risk factor and a stage in the suicidal process from planning to attempting to dying [18,19]. A 60% rate of transition has been observed from suicidal ideation to first suicide attempt within the first year of ideation onset [19]. Hence, one of the key challenges for preventing suicide among adolescents is improving the prediction of suicide risk [20]. In Brazil, the prevalence of suicidal ideation and suicide attempt observed to be 14.0% and 5.9%, respectively, and associated with violent behavior, smoking and alcohol consumption [21]. Bullying of students was found to be common in Chile and associated with suicidal ideation [22]. Family problems, social pressure, economic struggle, racism and violence introduce constant emotional stresses and challenges among Peruvian adolescents [23]. In addition, a strong association was observed between peer victimization and emotional and mental stress among adolescents in Peru [24]. However, suicidal behavior and its associated factors among adolescents in Peru are unknown. Therefore, the objective of the current study was to identify the prevalence of suicidal ideation and suicide attempts, as well as associated demographic, socio-environmental, psychological and behavioral factors of suicidal ideation and suicide attempt. In the study, we tried to analyze attributes of suicidal ideation in three different models, model 1 comprised of demographic and socio-environmental variables, model 2 psychological and behavioral variables, and model 3 consisted of all study variables. This study provides useful information regarding suicidal ideation and suicide attempt, and associated factors for concerned authorities and others who need it.

## 2. Methods

### 2.1. Study Design, Population and Sampling

The information for the study was collected as a part of school heath survey among secondary school students. The field study was conducted from 15 September to 31 October 2014 collaborating with Korea International Cooperation Agencies (KOICA), Peru office. This was a cross-sectional survey conducted in one district of Lima (Comas) and two districts of Callao (Bellavista and Ventanilla). These three districts in the Lima metropolitan area (Province of Lima and Callao) were purposively selected. From the three districts, six areas were randomly selected collaborating with National Institute of Statistics and Information, Peru. Three areas from Comas (Santa Luzmila II, Laura Rodriguez Dulanto and Carlos Philips), one area from Bellavista (Bellavista) and two areas from Ventanilla (Pachacutec and Mi Peru) were included for the survey. The target population was secondary school students. Out of 17 secondary schools in the study areas, 11 were randomly selected. Five secondary grade levels were considered as strata. One stratum from each school and students from each stratum were selected randomly. According to sample size calculation, the required sample size was 975. A total of 981 students were randomly selected and invited to participate questionnaire survey. Some students were absent on the day of information collection and some did not completed the questionnaire correctly. Thus, the collected sample size was 970. However, 54 samples were excluded from the analysis because of missing of dependent and some independent variables. As we have used low margin of error and non-response rate of 1.18, the sample size of 916 can adequately represent the study population. The detailed of sample size calculation has been put at appendix.

### 2.2. Data Collection and Measurement

An anonymous, self-administered structured questionnaire was adopted based on the Peruvian Student Health Survey (2010) and the Global School-based Student Health Survey [25,26]. Trained enumerators administered questionnaires to the randomly selected students in their classrooms during regular school hours. Before completing the questionnaire, a brief orientation was provided on the objective of the study and ways of providing responses, and they were encouraged to read the instructions carefully. The students were informed that the survey was anonymous. The teachers and school staffs were not allowed in the class room during information collection. Table 1 provides the information on the measurements of variables used in the study.

**Table 1:** Measurements of study variables, Peru, 2014.

Variables	Classification	Survey Questions
In fight	One or more times	During the past 12 months, how many times have you been in a physical fight?
	Never	
Insulted	One or more times	During the past 30 days, how many days have you received an insult?
	Never	
Attacked	Yes	During the past 12 months, were you physically attacked?
	No	
Parental understanding	Never/rarely/sometimes	During the past 30 days, how often did your parent or guardian try to understand your problems or worries?
	Most of the time/always	
Time spending with parents	Never/rarely/sometimes	During the past 30 days, how often did you spend time with your parent or guardian?
	Most of the time/always	
Parental homework checking	Never/rarely/sometimes	During the past 30 days, how often did your parent or guardian check to see if your homework was done?
	Most of the time/always	
School absenteeism	0–2 days	During the past 30 days, how many days did you miss class without giving notice to the school?
	≥3 days	
^a^ Perceived body weight satisfaction	Unsatisfied	What do you think about your body weight?
	Satisfied	
^b^ Self-rated health	Poor/fair health	What do you think about your health?
	Good health	
^c^ Perceived happiness	Unhappy	How do you feel about your life?
	Happy	
Smoking	Yes	During your life, have you ever smoked?
	No	
Alcohol consumption	Yes	During your life, have you ever drunk alcohol (excluding religious practices)?
	No	
Illicit drug use	Yes	During your life, have you used narcotics?
	No	
Sexual intercourse initiation	Yes	During your life, have you ever experienced sexual intercourse?
	No	
Forced sex	Yes	Have you ever been forced to have sexual relation?
	No	
Condom use	Yes	In your most recent experience of sexual intercourse, did you use a condom?
	No	
Suicide attempt	Yes	In the past 12 months, have you tried to end your life?
	No	
Psychological counselling	Yes	In the past 12 months, have you received psychological counseling?
	No	

Sources: Authors’ operationalization of variables based on literature review; ^a^ Perceived body weight: dissatisfied (“very underweight,” “slightly underweight,” “slightly overweight,” “overweight” or “very overweight”) and satisfied (“normal weight”); ^b^ Self -rated health: good health (“extremely healthy,” “very healthy” or “healthy”) and poor/fair health (“fair” or “bad”); ^c^ Perceived happiness: happy (“very happy,” “happy” or “a bit happy”) and unhappy (“a bit sad,” “sad” or “very sad”).

### 2.3. Statistical Analysis

The sample data were entered and analyzed using SPSS for Windows, version 21 (IBM Corp.: Armonk, NY, USA). The characteristics of the study population and prevalence of suicidal ideation and attempt were presented as frequencies. To determine the factors contributing to suicidal ideation and suicide attempt, univariate and multivariate logistic regression analysis was conducted. The factors that were significant at the 5% level in univariate analysis were included in multivariate analysis. Three models were developed: the first comprised demographic and socio-environmental factors, the second comprised psychological and behavioral factors and the third comprised all of the factors that were significant in models 1 and 2. The analysis in three different models was intended to see the influence of personal (psychological and behavioral), non-personal factors, and integrated influence of all factors. Unadjusted and adjusted odds ratios (AORs) were presented with the 95% confidence intervals (CIs) and *p* values. The Hosmer and Lemeshow test was applied to determine the goodness of fit of the models, and all three models had a good fit with the observed values.

### 2.4. Ethical Considerations

Ethical approval for this study was obtained from the Institutional Review Board of Wonju Campus, Yonsei University (1041849-201410-BM-048-02) and the DIRESA Callao (local government of Peru). Prior consent was obtained from each school administration and parents or guardians. Informed assent was obtained from individual participants and an anonymous questionnaire was used.

## 3. Results

Table 2 presents the characteristics of the study population and the prevalence of suicidal ideation and attempt during the past 12 months. In the study sample of 916 students, 53.6% were girls, 53.1% were in the age group of 15–18 years and the median age was 15 years. Of the study participants, 34.2% had been involved in fighting, 41.3% had been insulted, 26.5% had been physically attacked and 17.9% had been physically attacked by their own family members during the 12 months preceding the survey. Regarding parental support, 49.8% of the participants reported that their parents did not spend time frequently with them, 64.8% that their parents did not understand their problems and 65.0% that their parents did not check their homework regularly. Regarding the psychological factors, 9% of participants reported being unhappy with their lives, 45.2% were unsatisfied with their body weight and 33.0% felt they had poor/fair health status. Regarding health risk behaviors, 25.3% had smoked, 49.2% had drunk alcohol, 7.0% had taken illicit drugs and 19.4% had had sexual intercourse. Among those who ever had sexual intercourse, 9.7% had experience of forced sexual relation, 51.7% did not use condom at their last sexual intercourse and 52.3% had multiple sexual partners. The prevalence of suicidal ideation and suicide attempt were 26.3% and 17.5%, respectively in the past 12 months. Of total, 13.8% of respondents; and of those who had reported suicidal ideation and suicide attempt, 19.0% and 21.2%, respectively, had received psychological counseling in the past 12 months.

**Table 2:** Characteristics of the study population and prevalence of suicidal ideation and suicide attempt, Peru, 2014.

Variables	Number	%
**Demographic Factors**		
Sex		
Female	491	53.6
Male	425	46.4
Age (in years)		
12–14	430	46.9
15–18	486	53.1
**Socio-Environmental Factors**		
In fight (*n* = 916)	313	34.2
Insulted (*n* = 896)	370	41.3
Attacked (*n* = 916)	243	26.5
Family member as physical attacker (*n* = 916)	164	17.9
≥3 days school absenteeism (*n* = 903)	76	8.4
Parental understanding (*n* = 898)		
Never/rarely/sometimes	582	64.8
Spending time with parents (*n* = 898)		
Never/rarely/sometimes	447	49.8
Parental homework checking (*n* = 902)		
Never/rarely/sometimes	586	65.0
**Psychological Factors**		
Body weight dissatisfaction (*n* = 916)	414	45.2
Self-rated poor/fair health (*n* = 916)	302	33.0
Perceived unhappiness (*n* = 916)	82	9.0
**Behavioral Factors**		
Smoking (*n* = 909)	230	25.3
Alcohol consumption (*n* = 896)	441	49.2
Illicit drugs use (*n* = 896)	63	7.0
Sexual intercourse (*n* = 907)	176	19.4
Forced sex (*n* = 176)	17	9.7
Condom use (*n* = 176)	91	51.7
Multiple sexual partners (*n* = 176)	92	52.3
**Suicidal Behaviors**		
Suicidal ideation (*n* = 916)	241	26.3
Suicide attempt (*n* = 916)	160	17.5
Psychological counseling (*n* = 916)	126	13.8

Source: Authors’ calculation.

Table 3 shows the factors associated with suicidal ideation in unadjusted and adjusted models. In the unadjusted model, all variables except age group were significantly associated with dependent variable. In model 1, while gender and all socio-environmental variables adjusted simultaneously, school absenteeism and parental homework checking did not remain significant. Thus, female sex, being in a fight, being insulted, being attacked, lack of parental understanding and less time spending with parents were statistically significant factors socio-demographic of suicidal ideation. In the model 1, Nagelkerke *R*^2^ value indicates that 28.8% of suicidal ideation variance is explained by demographic and socio-environmental factors. Out of two psychological and four behavioral factors adjusted in model 2, two psychological—self-rated poor/fair health, perceived unhappiness—and three behavioral factors—smoking, alcohol consumption and sexual intercourse initiation—were statistically significant factors of suicidal ideation. In model 3, all significant factors of model 1 and model 2 were entered simultaneously, comprising six factors of model 1 and five factors of model 2. However, parental understanding and spending time with parents that were significant in model 1, and poor/fair self-rated health and alcohol consumption of model 2 were not found significant with suicidal ideation. Finally, in model 3, female sex (AOR, 5.12; CI, 3.32–7.89), being in fight (AOR, 1.80; CI, 1.20–2.71), being insulted (AOR, 2.31; CI, 1.60–3.34), being attacked (AOR, 2.09; CI, 1.41–3.10), perceived unhappiness (AOR, 2.36; CI, 1.32–4.24), smoking (AOR, 1.70; CI, 1.08–2.66) and ever having sexual intercourse (AOR, 1.84; CI, 1.15–2.95) were significantly associated with suicidal ideation. In the final model, value of Nagelkerke *R*^2^ = 0.34, meaning that 34.0% of suicidal ideation variance is explained by the factors of model 3.

**Table 3:** Variables associated with suicidal ideation among school-going adolescents, Peru, 2014.

Variables	Unadjusted OR (95% CI)	Adjusted OR (95% CI)		
		Model 1	Model 2	Model 3
**Demographic Factors**				
Female sex	3.18 (2.30–4.39) ^a^	4.53 (3.08–6.68) ^a^		5.12 (3.32–7.89) ^a^
12–14 years age	1.09 (0.81–1.47)	-		-
**Socio-Environmental Factors**				
In fight	1.77 (1.31–2.40) ^a^	1.81(1.23–2.65) ^b^		1.80 (1.20–2.71) ^b^
Insulted	3.23 (2.37–4.39) ^a^	2.46 (1.73–3.49) ^a^		2.31 (1.60–3.34) ^a^
≥3 Days school absenteeism	2.07 (1.28–3.36) ^b^	0.64 (0.35–1.15)		-
Attacked	3.33 (2.43–4.57) ^a^	2.71 (1.87–3.92) ^a^		2.09 (1.41–3.10) ^a^
Parental understanding				
Never/rarely/sometimes	2.42 (1.71–3.42) ^a^	1.93 (1.27–2.94) ^b^		1.47 (0.96–2.27)
Spending with parents				
Never/rarely/sometimes	2.35 (1.72–3.20) ^a^	1.54 (1.05–2.25) ^c^		1.36 (0.92–2.02)
Parental homework checking				
Never/rarely/sometimes	1.50 (1.09–2.08) ^c^	0.85 (0.57–1.26)		
**Psychological Factors**				
Body weight dissatisfaction	0.861 (0.72–1.02)		-	-
Self-rated poor/fair health	2.21 (1.63–3.00) ^a^		1.80 (1.28–2.53) ^b^	1.27 (0.86–1.87)
Perceived unhappiness	4.96 (3.10–7.94) ^a^		3.77 (2.27–6.28) ^a^	2.36 (1.32–4.24) ^b^
**Behavioral Factors**				
Smoking	2.47 (1.79–3.41) ^a^		1.64 (1.10–2.44) ^c^	1.70 (1.08–2.66) ^c^
Alcohol consumption	2.33 (1.71–3.17) ^a^		1.65 (1.15–2.38) ^b^	1.41 (0.94–2.10)
Illicit drug use	2.40 (1.42–4.04) ^b^		1.54 (0.84–2.83)	-
Sexual intercourse initiation	2.37 (1.67–3.36) ^a^		1.57 (1.03–2.38) ^c^	1.84 (1.15–2.95) ^c^
Nagelkerke *R*^2^		0.288	0.157	0.340
* *p* value		0.297	0.853	0.093

Source: Authors’ calculation, ^a^ *p* < 0.001; ^b^ *p* < 0.01; ^c^ *p* < 0.05; * Hosmer and Lemeshow Test.

Table 4 shows that factors associated with suicide attempt among study population. In the unadjusted model, all variables except age group were significantly associated with suicide attempt, as they were with suicidal ideation. In the model 1, while gender and all socio-environmental variables adjusted simultaneously, school absenteeism and parental homework checking did not remain significant with suicide attempt. The value of Nagelkerke shows that 24.3% of suicide attempt variance is explained by demographic and socio-environmental factors.

**Table 4:** Variables associated with suicide attempt among school-going adolescents, Peru, 2014.

Variables	Unadjusted OR (95% CI)	Adjusted OR (95% CI)		
		Model 1	Model 2	Model 3
**Demographic factors**				
Female sex	3.36 (2.27–4.97) ^a^	4.51 (2.88–7.06) ^a^		4.06 (2.53–6.52) ^a^
12–14 years age	0.94 (0.67–1.32)	-		-
**Socio-environmental factors**				
In fight	1.74 (1.23–2.47) ^b^	1.71 (1.12–2.60) ^c^		1.78 (1.14–2.77) ^c^
Insulted	3.37 (2.34–4.84) ^a^	2.55 (1.71–3.81) ^a^		2.39 (1.57–3.64) ^a^
≥3 days school absenteeism	0.52 (0.30–0.88) ^c^	0.70 (0.37–1.33)		-
Attacked	2.98 (2.09–4.26) ^a^	2.18 (1.45–3.27) ^a^		1.87 (1.21–2.89) ^b^
Parental understanding				
Never/rarely/sometimes	2.42 (1.60–3.67) ^a^	1.70 (1.05–2.78) ^c^		1.38 (0.84–2.28)
Time spending with parents				
Never/rarely/sometimes	2.46 (1.70–3.55) ^a^	1.60 (1.04–2.47) ^c^		1.38 (0.88–2.17)
Parental homework checking				
Never/rarely/sometimes	1.67 (1.13–2.46) ^b^	0.94 (0.59–1.48)		-
**Psychological factors**				
Body weight dissatisfaction	1.47 (1.04–2.07) ^c^		1.19 (0.81–1.74)	-
Self-rated poor/fair health	2.40 (1.70–3.40) ^a^		1.88 (1.27–2.78) ^b^	1.29 (0.84–1.98)
Perceived unhappiness	5.34 (3.32–8.58) ^a^		4.05 (2.40–6.83) ^a^	2.72(1.51–4.88) ^b^
**Behavioral factors**				
Smoking	2.07 (1.43–2.98) ^a^		1.41 (0.89–2.22)	-
Alcohol consumption	2.28 (1.58–3.27) ^a^		1.59 (1.04–2.44) ^c^	1.52 (1.00–2.33) ^c^
Illicit drug use	3.02 (1.75–5.22) ^a^		2.37 (1.24–4.51) ^b^	2.91 (1.51–5.61) ^b^
Sexual intercourse initiation	2.17 (1.47–3.20) ^a^		1.30 (0.80–2.10)	-
Nagelkerke R^2^		0.243	0.155	0.290
* *p* value		0.366	0.282	0.361

Source: Authors’ calculation, ^a^ *p* < 0.001; ^b^ *p* < 0.01; ^c^ *p* < 0.05; * Hosmer and Lemeshow Test.

In this model, female sex, being in a fight, being insulted, being attacked, lack of parental understanding and less time spending with parents were statistically associated with suicide attempt. Out of three psychological and four behavioral factors adjusted in model 2, two psychological—self-rated poor/fair health, perceived unhappiness—and two behavioral factors—alcohol consumption and illicit drug use—were significantly related with suicide attempt. In model 3, all significant factors of model 1 and model 2 were entered simultaneously, comprising six factors of model 1 and four factors of model 2. Two socio-environmental factors—parental understanding and spending time with parents—and one psychological factor—poor/fair self-rated health—became insignificant factors with suicide attempt. Finally, in model 3, female sex (AOR, 4.06; CI, 2.53–6.52), being in fight (AOR, 1.78; CI, 1.14–2.77), being insulted (AOR, 2.39; CI, 1.57–3.64), being attacked (AOR, 1.87; CI, 1.21–2.89), perceived unhappiness (AOR, 2.72; CI, 1.51–4.88), alcohol consumption (AOR, 1.52; CI, 1.00–2.33) and illicit drug use (AOR, 2.91; CI, 1.51–5.61) were significantly associated with increased likelihood of suicide attempts. Considering the value of Nagelkerke *R*^2^, 29.0% of suicide attempt variance is explained by model 3.

## 4. Discussion

The study shows that a large proportion of adolescents in Peru reported having suicidal ideation and having attempted suicide during the prior 12 months. A large number of factors; demographic, socio-environmental, psychological and behavioral factors are significantly associated with increased risk of suicidal ideation and suicide attempts in Peru. In this study, the prevalence of suicidal ideation and suicide attempt was 26.3% and 17.5%, respectively, which is relatively high when considering similar studies in various parts of the world [27,28,29,30].

In the current study, female were more likely than male to have suicidal ideation and attempt suicide. Other studies have also observed significantly higher suicidal ideation [14,21,29,30,31,32,33] and higher suicide attempts [21,31,34] among females. A study explains that girls had higher suicide attempt risk than boys, and 45% of the risk is explained by socioeconomic, school and mental difficulties and violence [35]. Regarding the age variation, as in other studies [14,21,33], the study also did not identify any association between age group and suicidal ideation and suicide attempt which indicate equal risk of suicidal ideation and suicide attempt among different aged adolescents.

In the study, being in fights, being physically attacked and being insulted were associated with increased occurrence of suicidal ideation as well as suicide attempt among school-going adolescents. Students involved in fights were more like to think seriously about committing suicide. In the study samples, 34.2% involved in fight, 41.3% were insulted and 26.5% were physically attacked in the past one year which indicates adolescent violence and bullying seemed to be common in study area. Among Brazilian adolescents also, being involved in fight was associated with a higher rate of suicidal ideation and suicide attempt [21]. Similarly, being bullied has been seen as an important risk factor of suicidal ideation and attempt in previous findings in other countries [14,28,29,33]. This may show physical and emotional violence against adolescents is related with suicidal ideation and suicide attempt. Thus, violence against adolescents and fighting between adolescents should be addressed to prevent suicidal behaviors in Peru. However, the study did not assess sexual violence among study population and its association with suicidal ideation and attempt. In the study, problem understanding by parents and spending time with parents were significant socio-environmental factors for suicidal ideation and suicide attempt in model 1, but both were insignificant in model 3. However, other studies have shown that parental support reduces the risk of suicidal ideation [14,28,29] and suicide attempt [36]. A study involving Latino adolescents points out the importance of family involvement for suicide prevention programs [36].

The study revealed a statistically significant positive association between smoking and suicidal ideation. Similarly, a number of earlier studies also observed cigarette smoking as risk factor of suicidal ideation [12,15,21,37] and suicide attempts [21]. However, we did not find a statistically significant association between smoking and suicide attempts in the study. Regarding alcohol consumption, it was significantly linked with suicide attempt, but with suicide ideation only in model 2. As reported by other studies, alcohol consumption has frequently been found to be related to suicidal ideation [14,17,28,38] and attempted suicide [21,28]. We also found significant associated between use of illicit drug and suicide attempt in the study. Likewise, a large number of studies have found strong associations between illicit drug use and suicidal ideation and suicide attempt [14,27,28]. Particularly early initiation of smoking, alcohol and drug are strongly associated with adolescent suicidal ideation and attempt [39,40]. In the study sample, experience of smoking, alcohol and illicit drug use was 25.3%, 49.2% and 7.0% respectively. Thus, preventing initiation of smoking, alcohol and illicit drugs among adolescents can be one of the useful strategies to prevent suicide in Peru.

Some studies observed that adolescents who have initiated sexual intercourse are more likely to have suicidal ideation and attempt suicide [16,40]. In the current study, we found experience of sexual intercourse was significantly associated with suicidal ideation. However, significant association was not found between sexual intercourse and suicide attempt. In Peru, about one-fifth of the adolescent aged 13-18 years had already initiated sexual intercourse [41]. In the study sample, 19.2% ever had experience of sexual intercourse, and among them, 9.7% had experience of forced sexual relations, 51.7% did not use a condom during their last sexual intercourse. The non-use of condom and contraception make adolescents vulnerable to unwanted pregnancy and sexually transmitted infections [42]. Thus, prevention of early sexual initiation and promotion of safe sex behavior may improve adolescent health, including suicide prevention.

Adolescents who were unhappy with their lives were more likely to report suicidal ideation and suicide attempts in the current study. Decreasing life satisfaction during adolescence can be understood as a development phenomenon, and should be considered when discussing the increasing prevalence of suicidal thoughts during adolescence [43]. Life dissatisfaction was observed as one of significant risk factors for suicidal ideation for both genders [44]. Perceived body weight dissatisfaction did not contribute to suicidal ideation or attempted suicide in the current study, although it was seen to be a strong risk factor in previous studies [45,46,47]. Female adolescents who perceived being overweight and male adolescents who perceived being underweight as well as overweight were more likely to report suicidal thought and suicide attempts [46]. A study among high school students concluded that perceived body weight may be more important than actual body mass index in term of increased likelihood of suicidal behavior [47].

The progression of suicidal phenomena from suicidal ideation to suicide attempt and suicide may not be always true [48]. Thus, factors that influence suicidal ideation and suicide attempts may be different. In the study, gender, violence-related factors and perceived unhappiness are associated to both ideation and suicide attempts. However, alcohol and illicit drug use are related only with suicide attempts and smoking and sexual intercourse initiation with suicidal ideation. 

Therefore, a number of psychological, behavioral, family and social factors influence the suicidal ideation and suicide attempts during adolescence that need to be considered while discussing adolescent mental health and suicide prevention.

## 5. Conclusions

The prevalence of suicidal ideation and suicide attempt was found relatively high and associated with a number of factors among school-going urban adolescents in the study area. Conclusively, being female, involvement in fights, being insulted, being attacked, perceived unhappiness are significantly associated with suicidal ideation as well as suicide attempts, while smoking and sexual intercourse initiation are associated only with suicidal ideation, and alcohol and illicit drug use are related only with suicide attempts. Therefore, suicidal behaviors prevention intervention is essential, and should focus on prevention of violence against adolescents, encouraging healthy peer relationships, promoting the supportive role of parents and controlling health risk behaviors, particularly initiation of smoking, alcohol and illicit drug use in adolescent.

## Appendix

### *Sample Size Calculation*

Samples from each school were taken using a proportionate simple random sampling technique. Total sample size was calculated by using the following formula:

m = [Z^2^ × V × M]/[d^2^ (M − 1) + Z^2^ ×V] × (def) × (tnr)

where, m: Students sample to be estimated, Z: Value of the normal distribution (Z = 1.96 for standard normal distribution at 95% confidence level), P: Prevalence of smoking and alcohol among school students (a study conducted in Peru showed that 23% students had tried smoking or alcohol within one year preceding the survey [49]) (P = 0.23), V: P × Q (Q = 1 – P= 0.77), M: Total number of students in the study area (M = 14,787), d: Margin of error (d = 0.0307), def: Clustering effect of the distribution of estimates (def = 1.2), tnr: Adjustment factors due to non-response ( tnr = 1.18). For the categorical variable, 5% margin of error is acceptable [50]. Higher value of margin of error yield smaller sample size.

## References

[1] World Health Organization (2014). Suicide. Fact Sheet 398.

[2] World Health Organization (2014). Preventing Suicide: A Global Imperative.

[3] Wasserman D., Cheng Q.I., Jiang G.X. (2005). Global suicide rates among young people aged 15–19. World Psychiatry.

[4] Patton G.C., Coffey C., Sawyer S.M., Viner R.M., Haller D.M., Bose K., Vos T., Ferguson J., Mathers C.D. (2009). Global patterns of mortality in young people: A systematic analysis of population health data. Lancet.

[5] Blum R.W. (2009). Young people: Not as healthy as they seem. Lancet.

[6] World Health Organization Mental Health Atlas-2011 Country Profiles.

[7] Devries K., Watts C., Yoshihama M., Kiss L., Schraiber L.B., Deyessa N., Heise L., Durand J., Mbwambo J., Jansen H. (2011). Violence against women is strongly associated with suicide attempts: Evidence from the WHO multi-country study on women’s health and domestic violence against women. Soc. Sci. Med..

[8] Miller A.B., Esposito-Smythers C., Weismoore J.T., Renshaw K.D. (2013). The relation between child maltreatment and adolescent suicidal behavior: A systematic review and critical examination of the literature. Clin. Child Fam. Psychol. Rev..

[9] Enns M.W., Cox B.J., Afifi T.O., de Graaf R., Ten Have M., Sareen J. (2006). Childhood adversities and risk for suicidal ideation and attempts: A longitudinal population-based study. Psychol. Med..

[10] Martin G., Bergen H.A., Richardson A.S., Roeger L., Allison S. (2004). Sexual abuse and suicidality: Gender differences in a large community sample of adolescents. Child Abuse Negl..

[11] Joiner T.E., Sachs-Ericsson N.J., Wingate L.R., Brown J.S., Anestis M.D., Selby E.A. (2007). Childhood physical and sexual abuse and lifetime number of suicide attempts: A persistent and theoretically important relationship. Behav. Res. Ther..

[12] Garrison C.Z., McKeown R.E., Valois R.F., Vincent M.L. (1999). Aggression, substance use, and suicidal behaviors in high school students. Am. J. Public Health.

[13] Afifi T.O., Cox B.J., Katz L.Y. (2007). The association between health risk behavior and suicidal ideation and attempt in a nationally representative sample of young adolescents. Can. J. Psychiatr..

[14] Randall J.R., Doku D., Wilson M.L., Peltzer K. (2014). Suicidal behaviour and related risk factors among school-aged youth in the Republic of Benin. PLoS ONE.

[15] Han M.A., Kim K.S., Ryu S.Y., Kang M.G., Park J. (2009). Associations between smoking and alcohol drinking and suicidal behavior in Korean adolescents: Korea Youth Behavioral Risk Factor Surveillance, 2006. Prev. Med..

[16] Peltzer K., Pengpid S. (2012). Suicidal ideation and associated factors among school-going adolescents in Thailand. Int. J. Environ. Res. Public Health.

[17] Muula A.S., Kazembe L.N., Rudatsikira E., Siziya S. (2007). Suicidal ideation and associated factors among in-school adolescents in Zambia. Tanzan. Health Res. Bull..

[18] Thanh H.T., Tran T.N., Jiang G.X., Leenaars A., Wasserman D. (2006). Life time suicidal thoughts in an urban community in Hanoi, Vietnam. BMC Public Health.

[19] Nock M.K., Borges G., Bromet E.J., Alonso J., Angermeyer M., Beautrais A., Bruffaerts R., Chiu W.T., de Girolamo G., Gluzman S. (2008). Cross-national prevalence and risk factors for suicidal ideation, plans and attempts. Br. J. Psychiatr..

[20] Hawton K., Saunders K.E., O’Connor R.C. (2012). Self-harm and suicide in adolescents. Lancet.

[21] Silva R.J.D.S., Santos F.A.L.D., Soares N.M.M., Pardono E. (2014). Suicidal ideation and associated factors among adolescents in northeastern Brazil. Sci. World J..

[22] Fleming L.C., Jacobsen K.H. (2009). Bullying and symptoms of depression in Chilean middle school students. J. Sch. Health.

[23] Bayer A.M., Gilman R.H., Tsui A.O., Hindin M.J. (2010). What is adolescence?: Adolescents narrate their lives in Lima, Peru. J. Adolesc..

[24] Lister C.E., Merrill R.M., Vance D.L., West J.H., Hall P.C., Crookston B.T. (2015). Victimization among Peruvian adolescents: Insights into mental/emotional health from the Young Lives study. J. Sch. Health.

[25] Centre for Disease Control and Prevention (2010). Global School-Based Student Health Survey (GSHS).

[26] World Health Organization (2013). Global School-Based Student Health Survey (GSHS), Questionnaire Modules.

[27] Chen P.C., Lee L.K., Wong K.C., Kaur J. (2005). Factors relating to adolescent suicidal behavior: A cross-sectional Malaysian school survey. J. Adolesc. Health.

[28] Mahfoud Z.R., Afifi R.A., Haddad P.H., DeJong J. (2011). Prevalence and determinants of suicide ideation among Lebanese adolescents: Results of the GSHS Lebanon 2005. J. Adolesc..

[29] Rudatsikira E., Muula A.S., Siziya S. (2007). Prevalence and associated factors of suicidal ideation among school-going adolescents in Guyana: Results from a cross sectional study. Clin. Pract. Epidemiol. Ment. Health.

[30] Liu X., Tein J.Y., Zhao Z., Sandler I.N. (2005). Suicidality and correlates among rural adolescents of China. J. Adolesc. Health.

[31] Bhola P., Rekha D.P., Sathyanarayanan V., Daniel S., Thomas T. (2014). Self-reported suicidality and its predictors among adolescents from a pre-university college in Bangalore, India. Asian J. Psychiatr..

[32] Delfabbro P.H., Winefield H.R., Winefield A.H. (2013). Life-time and current suicide-ideation in Australian secondary school students: Socio-demographic, health and psychological predictors. J. Affect. Disord..

[33] Rudatsikira E., Muula A.S., Siziya S., Twa-Twa J. (2007). Suicidal ideation and associated factors among school-going adolescents in rural Uganda. BMC Psychiatr..

[34] Kang E.H., Hyun M.K., Choi S.M., Kim J.M., Kim G.M., Woo J.M. (2015). Twelve-month prevalence and predictors of self-reported suicidal ideation and suicide attempt among Korean adolescents in a web-based nationwide survey. Aust. N. Z. Psychiatr..

[35] Chau K., Kabuth B., Chau N. (2014). Gender and family disparities in suicide attempt and role of socioeconomic, school, and health-related difficulties in early adolescence. Biomed. Res. Int..

[36] Kuhlberg J.A., Pena J.B., Zayas L.H. (2010). Familism, parent-adolescent conflict, self-esteem, internalizing behaviors and suicide attempts among adolescent Latinas. Child Psychiatr. Hum. Dev..

[37] Wilson M.L., Dunlavy A.C., Viswanathan B., Bovet P. (2012). Suicidal expression among school-attending adolescents in a middle-income sub-Saharan country. Int. J. Environ. Res. Public Health.

[38] Swahn M.H., Bossarte R.M. (2007). Gender, early alcohol use, and suicide ideation and attempts: Findings from the 2005 youth risk behavior survey. J. Adolesc. Health.

[39] Peltzer K., Pengpid S. (2015). Early substance use initiation and suicide ideation and attempts among school-aged adolescents in four Pacific Island Countries in Oceania. Int. J. Environ. Res. Public Health.

[40] Kim D.S., Kim H.S. (2010). Early initiation of alcohol drinking, cigarette smoking, and sexual intercourse linked to suicidal ideation and attempts: Findings from the 2006 Korean Youth Risk Behavior Survey. Yonsei Med. J..

[41] Osorio A., Lopez-del Burgo C., Ruiz-Canela M., Carlos S., de Irala J. (2015). Safe-sex belief and sexual risk behaviours among adolescents from three developing countries: A cross-sectional study. BMJ Open.

[42] Gonçalves H., Machado E.C., Soares A.L.G., Camargo-Figuera F.A., Seering L.M., Mesenburg M.A., Guttier M.C., Barcelos R.S., Buffarini R., Assunção M.C.F. (2015). Sexual initiation among adolescents (10 to 14 years old) and health behaviors. Rev. Bras. Epidemiol..

[43] Goldbeck L., Schmitz T.G., Besier T., Herschbach P., Henrich G. (2007). Life satisfaction decreases during adolescence. Qual. Life Res..

[44] Park H.S., Koo H.Y., Schepp K.G. (2005). Predictors of suicidal ideation for adolescents by gender. Taehan Kanho Hakhoe Chi.

[45] Kim D.S. (2009). Body image dissatisfaction as an important contributor to suicidal ideation in Korean adolescents: Gender difference and mediation of parent and peer relationships. J. Psychosom. Res..

[46] Whetstone L.M., Morrissey S.L., Cummings D.M. (2007). Children at risk: The association between perceived weight status and suicidal thoughts and attempts in middle school youth. J. Sch. Health.

[47] Eaton D.K., Lowry R., Brener N.D., Galuska D.A., Crosby A.E. (2005). Associations of body mass index and perceived weight with suicide ideation and suicide attempts among US high school students. Arch. Pediatr. Adolesc. Med..

[48] Sveticic J., de Leo D. (2012). The hypothesis of a continuum in suicidality: A discussion on its validity and practical implications. Ment. Ill.

[49] DEVIDA-Comision Nacional Para El Desarrollo Y Vida Sin Drogas (2013). IV Estudio Nacional: Prevención y Consumo de Drogas en Estudiantes de Secundaria 2012.

[50] Krejcie R.V., Morgan D.W. (1970). Determining sample size for research activities. Educ. Psychol. Meas..

